# Supplementary material for: Aging power spectrum of membrane protein transport and other subordinated random walks
Source: Nat Commun. 2021 Oct 25;12:6162. doi: 10.1038/s41467-021-26465-8 (PMC8546023; doi:10.1038/s41467-021-26465-8)
Supplement: Supplementary file 1 — Supplementary Information [file 41467_2021_26465_MOESM1_ESM.pdf]

Supplementary Information: Aging power spectrum of membrane protein transport and other subordinated random walks

Zachary R Fox,[1,2] Eli Barkai,[3] and Diego Krapf[1,4]

[1] *School of Biomedical Engineering, Colorado State University, Fort Collins, Colorado 80523, U.S.A.*
[2] *The Center for Nonlinear Studies and Computational and Statistical Sciences Division, Los Alamos National Laboratory, Los Alamos, New Mexico 87545, U.S.A.*
[3] *Department of Physics, Institute of Nanotechnology and Advanced Materials, Bar Ilan University, Ramat-Gan 52900, Israel*
[4] *Electrical and Computer Engineering, Colorado State University, Fort Collins, Colorado 80523, U.S.A.*[*]

(Dated: September 27, 2021)

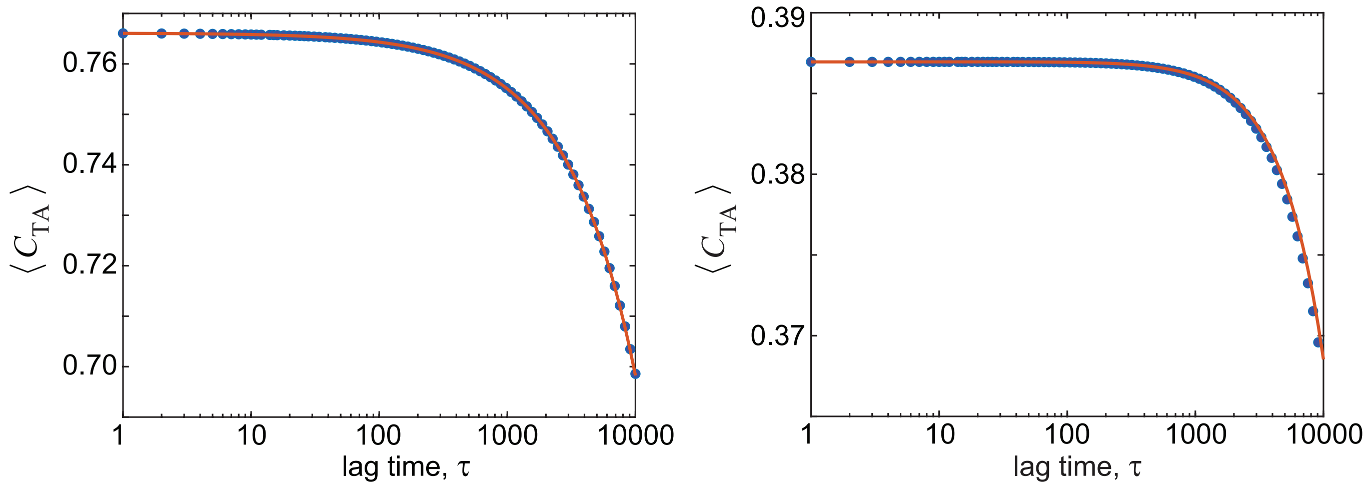

Supplementary Figure 1. Time average-autocorrelation function analytical results. (a) Subdiffusive fBM example, with Hurst exponent $H = 0.3$ and sojourn times with power law distribution with $\alpha = 0.4$. (b) Superdiffusive fBM example, with Hurst exponent $H = 0.7$ and sojourn times with power law distribution with $\alpha = 0.4$. Likely, the small deviations that show up when the process exhibits long-range dependence, i.e., $H > 1/2$, are due to the realization time not being long enough, In both datasets, the realization time is $t_{\mathrm{m}} = 2^{16}$ and the number of realizations is $10,000$. The solid lines show analytical results given by Eq. 27, where $\langle C_{\mathrm{TA}}(t_{\mathrm{m}}, \tau)\rangle = t_{\mathrm{m}}^{\gamma}\phi_{\mathrm{TA}}(\tau/t_{\mathrm{m}})$.

[*] diego.krapf@colostate.edu

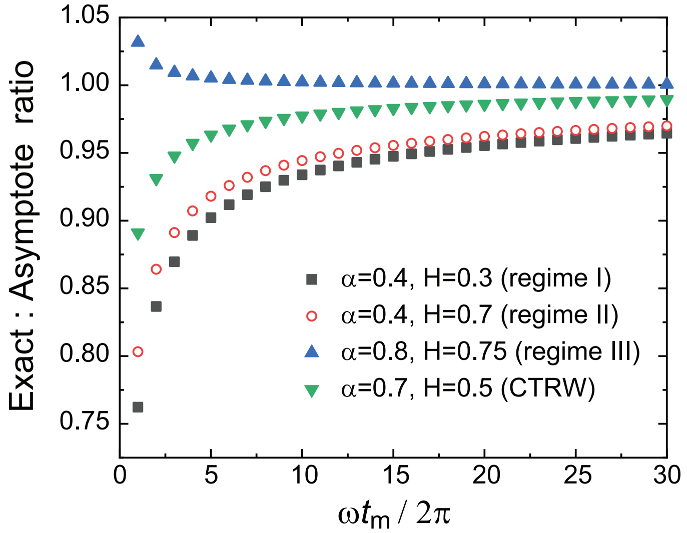

Supplementary Figure 2. Deviations between the exact results and the asymptotic approximations for the PSD. The deviations are presented for the four cases discussed in the text as the ratio between the exact hypergeometric function and the $1/\omega^{\beta}$ approximation for the lowest 30 natural frequencies.

SUPPLEMENTARY NOTE 1. POWER SPECTRAL DENSITY (PSD)

The PSD of a time-dependent signal $x(t)$ is defined as

$$\langle S(\omega,\infty)\rangle = \lim_{t_{\mathrm{m}}\to\infty}\frac{1}{t_{\mathrm{m}}}\left\langle\left|\int_0^{t_{\mathrm{m}}} e^{i\omega t}x(t)dt\right|^2\right\rangle, \tag{1}$$

where the angle brackets denote averaging over an infinitely large ensemble, i.e., the expected value. The Wiener Khinchin theorem provides a connection between the PSD and the autocorrelation function in stationary processes. Namely, this theorem states that the PSD is the Fourier transform of the autocorrelation function,

$$\langle S(\omega,\infty)\rangle = \int_{-\infty}^{\infty} e^{i\omega\tau}C(\tau)d\tau, \tag{2}$$

where $C(\tau)=\langle x(t)x(t+\tau)\rangle$ is the (ensemble-averaged) autocorrelation function.

SUPPLEMENTARY NOTE 2. FRACTIONAL MOMENTS OF THE NUMBER OF STEPS $\langle n^{\nu}(t)\rangle$ AND $\langle \Delta n^{\nu}(\tau;t)\rangle$

In order to solve the correlation function, we derive the fractional moments $\langle n^{\nu}(t)\rangle$ and $\langle \Delta n^{\nu}(\tau;t)\rangle$, where the relevant case for us is $\nu=2H$. The former is directly obtained from $\chi_n(t)$, the distribution of the number of steps up to time t, in the continuous approximation

$$\langle n^{\nu}(t)\rangle = \int_0^{\infty} n^{\nu}\chi_n(t)dn, \tag{3}$$

The distribution $\chi_n(t)$ is given by [1]

$$\chi_n(t) = \frac{t}{\alpha t_0}n^{-\frac{1}{\alpha}-1}L_{\alpha}\left(\frac{t}{n^{1/\alpha}t_0}\right), \tag{4}$$

where $L_{\alpha}(t)$ is the one sided Lévy function of order α. Then, Eq. 3 yields

$$\langle n^{\nu}(t)\rangle \approx \int_0^{\infty}\frac{t}{\alpha t_0}n^{\nu-\frac{1}{\alpha}-1}L_{\alpha}\left(\frac{t}{n^{1/\alpha}t_0}\right)dn. \tag{5}$$

The Laplace transform of the one-sided Lévy function is $\mathcal{L}\{L_{\alpha}(t)\}=\exp[-s^{\alpha}]$, thus the fractional moment of the number of steps in Laplace domain is

$$\begin{aligned}\langle \widetilde{n^{\nu}}(s)\rangle &\approx \int_0^{\infty} t_0^{\alpha}n^{\nu}s^{\alpha-1}\exp\left(-nt_0^{\alpha}s^{\alpha}\right)dn\\ &\approx \frac{\nu(1+\nu)}{t_0^{\alpha\nu}s^{1+\alpha\nu}}.\end{aligned} \tag{6}$$

Finally, we can use Tauberian theorem [1] to obtain the inverse Laplace transform,

$$\langle n^{\nu}(t)\rangle \approx \frac{\Gamma(1+\nu)}{t_0^{\alpha\nu}\Gamma(1+\alpha\nu)}t^{\alpha\nu}. \tag{7}$$

Along the same lines, the second term in Eq. 20 is the fractional moment

$$\langle n^{\nu}(t+\tau)\rangle \approx \frac{\Gamma(1+\nu)}{t_0^{\alpha\nu}\Gamma(1+\alpha\nu)}(t+\tau)^{\alpha\nu}. \tag{8}$$

In order to solve for the third term in the ACF, the fractional moment $\langle\Delta n^{\nu}(\tau;t_a)\rangle$ is obtained by considering Eq. 7 and the forward waiting time t_F for the first step of CTRW after the aging time t_a. The distribution of forward waiting times is [1]

$$\psi_1(t_F;t_a) = \frac{\sin(\pi\alpha)}{\pi}\left(\frac{t_a}{t_F}\right)^{\alpha}\frac{1}{t_a+t_F}, \tag{9}$$

so that

$$\begin{aligned}\langle \Delta n^{\nu}(\tau;t_a)\rangle &= \int_0^{\tau} \langle n^{\nu}(\tau - t_F)\rangle \psi_1(t_F;t_a)dt_F \\ &= \frac{\sin(\pi\alpha)}{\pi}\frac{\Gamma(1+\nu)}{t_0^{\alpha\nu}\Gamma(1+\alpha\nu)}\int_0^{\tau}\frac{t_a^{\alpha}(\tau-t_F)^{\alpha\nu}}{t_F^{\alpha}\,(t_a+t_F)}dt_F \\ &= \frac{\sin(\pi\alpha)}{\pi}\frac{\Gamma(1+\nu)\Gamma(1-\alpha)}{t_0^{\alpha\nu}\Gamma(2-\alpha+\alpha\nu)}{}_2F_1\left(1,1-\alpha;2-\alpha+\alpha\nu;-\frac{\tau}{t_a}\right)t_a^{\alpha-1}\tau^{1-\alpha+\alpha\nu}. \end{aligned} \tag{10}$$

The fractional moments $\langle n^{\nu}(t)\rangle$ and $\langle \Delta n^{\nu}(\tau;t)\rangle$ have been computed previously [2]. The solution for $\langle \Delta n^{\nu}(\tau;t)\rangle$ given in Eq. 25 of Ref. [2] has a different form but it is equivalent to Eq. 10 above.

In the limit where $\tau \ll t_a$, the distribution of forward waiting times becomes

$$\psi_1(t_F;t_a) = \frac{\sin(\pi\alpha)}{\pi}\frac{t_a^{\alpha-1}}{t_F^{\alpha}}, \tag{11}$$

so that

$$\begin{aligned}\langle \Delta n^{\nu}(\tau;t_a)\rangle &= \int_0^{\tau} \langle n^{\nu}(\tau - t_F)\rangle \psi_1(t_F;t_a)dt_F \\ &= \frac{\sin(\pi\alpha)}{\pi}\frac{\Gamma(1+\nu)t_a^{\alpha-1}}{t_0^{\alpha\nu}\Gamma(1+\alpha\nu)}\int_0^{\tau}\frac{(\tau-t_F)^{\alpha\nu}}{t_F^{\alpha}}dt_F \\ &= \frac{\sin(\pi\alpha)}{\pi}\frac{\Gamma(1+\nu)\Gamma(1-\alpha)}{t_0^{\alpha\nu}\Gamma(2-\alpha+\alpha\nu)}t_a^{\alpha-1}\tau^{1-\alpha+\alpha\nu}, \end{aligned} \tag{12}$$

which is also found directly from Eq. 10 because ${}_2F_1\left(1,1-\alpha;2-\alpha+\alpha\nu;0\right)=1$.

SUPPLEMENTARY NOTE 3. EXACT SOLUTIONS FOR THE AUTOCORRELATION FUNCTIONS AND PSD OF SUBORDINATED PROCESSES

A. Continuous time random walk ($2H = 1$)

We start with the solutions for a traditional CTRW [3, 4], i.e., $H = 0.5$. The ensemble-averaged autocorrelation function is

$$C_{\rm EA}(t,\tau) = \Delta x^2 \sum_{n=0}^{\infty}\sum_{\Delta n=0}^{\infty}\left(n^{2H} + (n+\Delta n)^{2H} - \Delta n^{2H}\right)\chi_{n,\Delta n}(t,\tau). \tag{13}$$

which, for the case $H = 0.5$, becomes

$$\begin{aligned} C_{\rm EA}(t,\tau) &= 2\Delta x \sum_{n=0}^{\infty}\sum_{\Delta n=0}^{\infty} n\chi_{n,\Delta n}(t,\tau) \\ &= 2\Delta x^2 \sum_{n=0}^{\infty} n\chi_n(t) = 2D\langle n(t)\rangle \\ &= 2\Delta x^2\langle n(t)\rangle, \end{aligned} \tag{14}$$

and, using Eq. 7, or the well know expression for the mean number of jumps in the interval $(0,t)$, $\langle n(t)\rangle$ at long times [1],

$$C_{\rm EA}(t,\tau) \sim \frac{2\Delta x}{t_0^{\alpha}\Gamma(1+\alpha)}t^{\alpha}. \tag{15}$$

The ensemble averaged autocorrelation function in Eq. 15 has the form $C_{\rm EA} = t^{\alpha}\phi_{\rm EA}$ and, thus, we obtain the time-averaged autocorrelation function

$$\langle C_{\rm TA}\rangle = t_{\rm m}^{\alpha}\phi_{\rm TA}(\tau/t_{\rm m}) \tag{16}$$

with the scaling function

$$\phi_{\mathrm{TA}}(y) = \frac{y^{1+\gamma}}{1-y}\int_{\frac{y}{1-y}}^{\infty}\frac{\phi_{\mathrm{EA}}}{z^{2+\gamma}}dz$$
$$= \frac{2\Delta x^2}{t_0^\alpha\Gamma(2+\alpha)}(1-y)^\alpha. \tag{17}$$

Using the time-averaged autocorrelation function in Eq. 17 and the aging Wiener-Khinchin theorem, we obtain the power spectral density of the CTRW,

$$\langle S_{\mathrm{CTRW}}(\omega, t_{\mathrm{m}})\rangle = 2t_{\mathrm{m}}^{1+\gamma}\int_0^1 (1-y)\phi_{\mathrm{TA}}(y)\cos(\omega t_{\mathrm{m}} y)dy.$$
$$= \frac{4\Delta x^2 t_{\mathrm{m}}^{1+\alpha}}{t_0^\alpha\Gamma(2+\alpha)}\int_0^1 (1-y)^{1+\alpha}\cos(\omega t_{\mathrm{m}} y)dy$$
$$= \frac{4\Delta x^2 t_{\mathrm{m}}^{1+\alpha}}{t_0^\alpha\Gamma(3+\alpha)}\,{}_1F_2\left[1;\frac{3+\alpha}{2},\frac{4+\alpha}{2};-\left(\frac{\omega t_{\mathrm{m}}}{2}\right)^2\right], \tag{18}$$

where ${}_1F_2\,(a;b_1,b_2;z)$ refers to the generalized hypergeometric function.

When $\alpha > 1$ the mean waiting time exists and the CTRW statistics revert in the long time to those of Brownian motion. In particular, replacing $\alpha = 1$ and $\omega t_{\mathrm{m}} = 2\pi k$ we find ${}_1F_2\left[1;2,5/2;-(\omega t_{\mathrm{m}})^2/4\right] = 6/(\omega t_{\mathrm{m}})^2$ and, thus, the PSD in Eq. 18 is that of standard Brownian motion,

$$\langle S_{\mathrm{BM}}(\omega)\rangle \sim \omega^{-2}, \tag{19}$$

which is independent of t_{m}.

B. Subordinated process involving fBM ($0 < H < 1$)

1. *Autocorrelation function*

When $H \neq 0.5$, the process has positively correlated increments for $H > 0.5$ and negatively correlated increments when $H < 0.5$. The autocorrelation function C_{EA} in Eq. 13 is

$$C_{\mathrm{EA}}(t,\tau) = \Delta x^2\left[\langle n^{2H}(t)\rangle + \langle n^{2H}(t+\tau)\rangle - \langle \Delta n^{2H}(\tau;t)\rangle\right]. \tag{20}$$

where $\Delta n(\tau;t)$ is the number of steps between the aged time t and $t+\tau$. From Eqs. 7, 8, and 10, the terms in Eq. 20 are found to be

$$\langle n^{2H}(t)\rangle = \frac{\Gamma(1+2H)}{t_0^\gamma\Gamma(1+\gamma)}t^\gamma, \tag{21}$$
$$\langle \Delta n^{2H}(\tau;t)\rangle = \frac{\Gamma(1+2H)}{t_0^\gamma\Gamma(1+\gamma)}b\;{}_2F_1\left(1,1-\alpha;2-\alpha+\gamma;-\frac{\tau}{t}\right)t^{\alpha-1}\tau^{1-\alpha+\gamma}, \tag{22}$$

where ${}_2F_1\,(a_1,a_2;b;z)$ is the Gaussian hypergeometric function. We have defined

$$\gamma = 2\alpha H, \tag{23}$$

and the constant b is

$$b = \frac{\sin(\pi\alpha)}{\pi}\frac{\Gamma(1-\alpha)\Gamma(1+\gamma)}{\Gamma(2-\alpha+\gamma)}. \tag{24}$$

Note that in the specific case that $H = 0.5$, these constants revert to $b = \gamma = \alpha$. Using a different formalism, $\langle \Delta n^\nu(\tau;t)\rangle$ has been previously derived [2, 5]. These previous results were expressed in terms of incomplete beta functions but they are equivalent to ours. The ensemble-averaged autocorrelation function, Eq. 20, is thus given by

$$C_{\mathrm{EA}}(t,\tau) = \Delta x^2 t^\gamma\left[1+\left(1+\frac{\tau}{t}\right)^\gamma - b\;{}_2F_1\left(1,1-\alpha;2-\alpha+\gamma;-\frac{\tau}{t}\right)\left(\frac{\tau}{t}\right)^{1-\alpha+\gamma}\right], \tag{25}$$

with

$$D=\frac{\Gamma(1+2H)}{\Gamma(1+\gamma)}\frac{\Delta x^2}{t_0^\gamma}, \tag{26}$$

which gives the EA-MSD when $\tau=0$; $\langle x^2(t)\rangle = 2\Delta x^2\langle n^{2H}(t)\rangle = 2Dt^\gamma$.

The ensemble-averaged autocorrelation function in Eq. 25 has the form $C_{\rm EA}(t,\tau)=t^\gamma\phi_{\rm EA}(\tau/t)$, which implies the time-averaged autocorrelation function is of the form $\langle C_{\rm TA}(t_{\rm m},\tau)\rangle = t_{\rm m}^\gamma\phi_{\rm TA}(\tau/t_{\rm m})$ [6]. Defining $y=\tau/t_{\rm m}$, we can find the scaling function (see Methods section in main text)

$$\phi_{\rm TA}(y)=\frac{D}{1+\gamma}\left[(1-y)^\gamma+\frac{1}{1-y}-\frac{(1+\gamma)b}{\alpha}\frac{y^{1+\gamma-\alpha}}{(1-y)^{1-\alpha}}{}_2F_1\left(1,-\alpha;2-\alpha+\gamma;-\frac{y}{1-y}\right)\right]. \tag{27}$$

Numerical simulations are observed to agree with analytical results for both $H<1/2$ and $H>1/2$ in Supplementary Figs. 1a and 1b, respectively.

2. Power spectral density

We see that the subordinated process with correlated increments shows that $\langle C_{\rm TA}\rangle = t_{\rm m}^\gamma\phi_{\rm TA}(\tau/t_{\rm m})$.The aging Wiener-Khinchin theorem gives the average power spectral density for the natural frequencies $\omega t_{\rm m}=2\pi k$ with k a non-negative integer,

$$\langle S(\omega,t_{\rm m})\rangle = 2t_{\rm m}^{1+\gamma}\int_0^1(1-y)\phi_{\rm TA}(y)\cos(\omega t_{\rm m}y)dy. \tag{28}$$

For the process subordinated to fractional Brownian motion, the time average autocorrelation function is found to be given by

$$\phi_{\rm TA}(y)=\frac{D}{1+\gamma}\left[(1-y)^\gamma+\frac{1}{1-y}-\frac{(1+\gamma)b}{\alpha}\frac{y^{1+\gamma-\alpha}}{(1-y)^{1-\alpha}}{}_2F_1\left(1,-\alpha;2-\alpha+\gamma;-\frac{y}{1-y}\right)\right]. \tag{29}$$

Therefore, to obtain $\langle S\rangle$, we compute the following three integrals with the notation $\tilde\omega=\omega t_{\rm m}$ and noting $\tilde\omega = 2\pi k$,

$$\int_0^1(1-y)^{1+\gamma}\cos(\tilde\omega y)dy=\frac{1}{2+\gamma}{}_1F_2\left[1;\frac{3+\gamma}{2},\frac{4+\gamma}{2};-\left(\frac{\tilde\omega}{2}\right)^2\right], \tag{30}$$

$$\int_0^1\cos(\tilde\omega y)dy=0, \tag{31}$$

$$\begin{aligned}&\int_0^1 y^{1-\alpha+\gamma}(1-y)^\alpha{}_2F_1\left(1,-\alpha;2-\alpha+\gamma;-\frac{y}{1-y}\right)\cos(\tilde\omega y)dy\\&=-(1-\alpha+\gamma)\frac{\Gamma(1+\alpha)\Gamma(2-\alpha+\gamma)}{\Gamma(3+\gamma)}{}_2F_3\left[\frac{2-\alpha+\gamma}{2},\frac{3-\alpha+\gamma}{2};\frac{3}{2},\frac{3+\gamma}{2},\frac{4+\gamma}{2};-\left(\frac{\tilde\omega}{2}\right)^2\right],\end{aligned} \tag{32}$$

where ${}_2F_3\left(a_1,a_2;b_1,b_2,b_3;z\right)$ is also a generalized hypergeometric function, leading to

$$\begin{aligned}\langle S(\omega,t_{\rm m})\rangle = 2Dt_{\rm m}^{1+\gamma}\Bigg[&\frac{1}{(1+\gamma)(2+\gamma)}{}_1F_2\left(1;\frac{3+\gamma}{2},\frac{4+\gamma}{2};-\left(\frac{\omega t_{\rm m}}{2}\right)^2\right)\\&+\frac{b(1-\alpha+\gamma)\Gamma(1+\alpha)\Gamma(2-\alpha+\gamma)}{\alpha\Gamma(3+\gamma)}{}_2F_3\left(\frac{2-\alpha+\gamma}{2},\frac{3-\alpha+\gamma}{2};\frac{3}{2},\frac{3+\gamma}{2},\frac{4+\gamma}{2};-\left(\frac{\omega t_{\rm m}}{2}\right)^2\right)\Bigg].\end{aligned} \tag{33}$$

SUPPLEMENTARY NOTE 4. PSD ASYMPTOTICS FOR LARGE $\omega t_{\rm m}$

The expansion of the hypergeometric functions for $\omega t_{\rm m} \gg 1$ (when $\omega t_{\rm m} = 2\pi k$) are

$$ {}_1F_2\left[1; \frac{3+\gamma}{2}, \frac{4+\gamma}{2}; -\left(\frac{\tilde{\omega}}{2}\right)^2\right] = (1+\gamma)(2+\gamma)(\omega t)^{-2} + \mathcal{O}(\omega t)^{-2-\gamma}, \tag{34} $$

$$ {}_2F_3\left[\frac{2-\alpha+\gamma}{2}, \frac{3-\alpha+\gamma}{2}; \frac{3}{2}, \frac{3+\gamma}{2}, \frac{4+\gamma}{2}; -\left(\frac{wt}{2}\right)^2\right] = \frac{\cos\left(\frac{\pi(\alpha-\gamma)}{2}\right)\Gamma(3+\gamma)}{(1-\alpha+\gamma)\Gamma(1+\alpha)}(\omega t)^{-2+\alpha-\gamma} + \mathcal{O}(\omega t)^{-2-\alpha}, \tag{35} $$

which leads to

$$ \langle S(\omega, t_{\rm m})\rangle \approx 2Dt_{\rm m}^{1+\gamma}\left[(\omega t_{\rm m})^{-2} + \frac{b\cos\left(\frac{\pi(\alpha-\gamma)}{2}\right)\Gamma(2-\alpha+\gamma)}{\alpha}(\omega t_{\rm m})^{-2+\alpha-\gamma}\right]. \tag{36} $$

Thus, the leading term for $\langle S(\omega, t_{\rm m})\rangle$ depends on the values of α and γ. In the case that $\alpha - \gamma > 0$,

$$ \langle S_{2H<1}(\omega, t_{\rm m})\rangle \approx 2ct_{\rm m}^{-(1-\alpha)}\omega^{-2+\alpha-\gamma}, \tag{37} $$

where

$$ c = \frac{Db}{\alpha}\cos\left(\frac{\pi(\alpha-\gamma)}{2}\right)\Gamma(2-\alpha+\gamma). \tag{38} $$

Note that $\gamma = 2\alpha H$ and thus $\alpha - \gamma > 0$ when $H < 0.5$, i.e., this is the leading term when the increments are anticorrelated. When the underlying fBM is superdiffusive (i.e, $H > 0.5$), $\alpha - \gamma < 0$ and the leading term is

$$ \langle S_{2H>1}(\omega, t_{\rm m})\rangle \approx 2Dt_{\rm m}^{-(1-\gamma)}\omega^{-2}. \tag{39} $$

SUPPLEMENTARY NOTE 5. QUANTIFICATION OF THE DEVIATIONS OF THE ASYMPTOTIC APPROXIMATIONS FROM THE EXACT PSD RESULTS

The asymptotic approximations of the PSD yield the familiar $1/\omega^\beta$ form. These approximations are very accurate in the limit $\omega t_{\rm m} \gg 1$. Thus, a fair question is how much do these approximations deviate from the exact results when the frequency is small? We evaluate the deviations at the small natural frequencies $\omega t_{\rm m} = 2\pi k$, with $k = 1, 2, 3, \ldots$. The results are presented for the four cases discussed in the manuscript in Supplementary Figure 2. Differences between the exact result (Equation 33) and the asymptotic approximations (Equations 37 and 39) are substantial only at the lowest natural frequencies. On a log-log plot, which is the common representation of $1/f$ type of spectra, these deviations are hard to detect.

---

[1] J. Klafter and I. M. Sokolov, First steps in random walks: from tools to applications (2011).

[2] J. H. Schulz, E. Barkai, and R. Metzler, Aging renewal theory and application to random walks, Phys. Rev. X **4**, 011028 (2014).

[3] E. W. Montroll and G. H. Weiss, Random walks on lattices. II, J. Math. Phys. **6**, 167 (1965).

[4] M. F. Shlesinger, Origins and applications of the Montroll-Weiss continuous time random walk, Eur. Phys. J. B **90**, 93 (2017).

[5] T. Akimoto and E. Barkai, Aging generates regular motions in weakly chaotic systems, Phys. Rev. E **87**, 032915 (2013).

[6] N. Leibovich, A. Dechant, E. Lutz, and E. Barkai, Aging Wiener-Khinchin theorem and critical exponents of $1/f^\beta$ noise., Phys. Rev. E **94**, 052130 (2016).
